# A randomized clinical trial to assess the effectiveness of pre- and post-surgical pelvic floor physiotherapy for bowel symptoms, pelvic floor function, and quality of life of patients with rectal cancer: CARRET protocol

**DOI:** 10.1186/s13063-021-05396-1

**Published:** 2021-07-13

**Authors:** Cinara Sacomori, Luz Alejandra Lorca, Mónica Martinez-Mardones, Roberto Ignacio Salas-Ocaranza, Guillermo Patricio Reyes-Reyes, Marta Natalia Pizarro-Hinojosa, Jorge Plasser-Troncoso

**Affiliations:** 1 Universidad Bernardo O’Higgins, Escuela de Kinesiología, Santiago, Chile; 2 Hospital del Salvador, Servicio de Medicina Física y Rehabilitación, Santiago, Chile; 3 Hospital del Salvador, Servicio de Cirugía y Servicio de Coloproctologia, Santiago, Chile; 4 Universidad Finnis Terrae, Escuela de Medicina, Santiago, Chile; 5 Fundación Arturo López Pérez, Santiago, Chile

**Keywords:** Rectal cancer, Pelvic floor, Low anterior resection syndrome, Rehabilitation, Prevention, Physiotherapy, Bowel symptoms, Quality of life

## Abstract

**Background:**

There is scarcity of trials about preventative strategies for low anterior resection syndrome (LARS) in rectal cancer patients. The aim of this study is to evaluate the effectiveness of a pre- and post-surgical pelvic floor rehabilitation program on the bowel symptoms, pelvic floor function, and quality of life of rectal cancer patients.

**Methods:**

A randomized controlled trial with parallel groups (pelvic floor rehabilitation versus control group), with a blinded evaluator. Participants and setting: 56 stage I to III rectal cancer patients aged from 18 to 80 years old undergoing sphincter preservation surgery at Hospital del Salvador and who have a sufficient knowledge of Spanish. Main outcome measures: ICIQ-B questionnaire for intestinal symptoms, high-resolution anorectal manometry (Alacer Multiplex 24-channel manometry equipment) for anorectal function, pelvic floor muscle strength test with Oxford Modified Scale, and a quality of life test with the EORTC QLQ C30 questionnaire. The evaluations will be carried out at five stages: before surgery, before and after the pelvic floor rehabilitation, and during a 3-month and 1-year follow-up. Interventions: one pre-rehabilitation session and 9 to 12 sessions of pelvic floor rehabilitation, including patient education, pelvic floor muscle exercises, pelvic floor electromyography biofeedback, and capacitive and sensory rectal training with a balloon probe. Rehabilitation will begin 3–5 weeks before the ileostomy is removed (four sessions) and around 3 weeks after stoma removal (5–8 sessions).

**Discussion:**

We expect the program to improve the bowel symptoms, pelvic floor function, and quality of life of rectal cancer patients.

**Trial registration:**

Australian New Zealand Clinical Trials Register ACTRN12620000040965. Registered on 21 January 2020.

## Administrative information

**Table:** 

Title {1}	A randomized clinical trial to assess the effectiveness of pre and post-surgical pelvic floor physiotherapy for bowel symptoms, pelvic floor function and quality of life of patients with rectal cancer: CARRET protocol
Trial registration {2a and 2b}.	ACTRN12620000040965. Australian New Zealand Clinical Trials Register
Protocol version {3}	First published version: 21/01/2020
Funding {4}	Government of Chile, Santiago de Chile. Fondo Nacional de Desarrollo Científico y Tecnológico [National Fund for Scientific and Technological Development], FONDECYT INICIACIÓN PROJECT award number 11191016, CONICYT/ANID [National Research and Development Agency]
Author details {5a}	Cinara Sacomori^1^*, Luz Alejandra Lorca^2^, Mónica Martinez-Mardones^3,4^, Roberto Ignacio Salas-Ocaranza^3^, Guillermo Patricio Reyes-Reyes^3^, Marta Natalia Pizarro-Hinojosa^2^, Jorge Plasser-Troncoso^3,5^ 1 Universidad Bernardo O´Higgins, Escuela de Kinesiología, Santiago de Chile, Chile. 2 Hospital del Salvador, Servicio de Medicina Física y Rehabilitación, Santiago de Chile, Chile. 3 Hospital del Salvador, Servicio de Cirugía y Servicio de Coloproctologia, Santiago de Chile, Chile. 4 Universidad Finnis Terrae, Escuela de Medicina, Santiago de Chile, Chile. 5 Fundación Arturo López Pérez, Santiago de Chile, Chile. *Corresponding author CS, LAL, MMM, RISO and GPRR initiated the study design and MNPH and JPT helped with implementation. MMM, RISO and JPT will help recruiting patients. MNPH will deliver the intervention. LAL and RISO will perform assessments. CS is granting holder and the main researcher and coordinator. All authors contributed to refinement of the study protocol and approved the final manuscript.
Name and contact information for the trial sponsor {5b}	A- Universidad Bernardo O´Higgins. Address: Avenida Viel 1497, Santiago, Region Metropolitana, Chile. B- Hospital del Salvador, Av. Salvador 364, Providencia, Región Metropolitana, Chile.
Role of sponsor {5c}	Funder and sponsors will not influence on study design, collection, management, analysis, and interpretation of data; writing of the report; and the decision to submit the report for publication.

## Introduction

### Background and rationale {6a}

Rectal cancer is a recognized health problem that compromises patients’ health-related quality of life. Worldwide in 2018, the incidence of rectal cancer was 3.9%, while its mortality rate was 3.2%, considering the total number of cancer cases [1]. Nevertheless, the prevalence of rectal cancer survivorship is increasing with the improvement of treatments, and surgery with radio-chemotherapies has significantly improved oncological outcomes [2]. In addition, the implementation of perioperative optimized protocol pathways has reduced rates of morbidity, improved recovery, and shortened the length of hospital stay [3].

Currently, surgeries for rectal cancer are less aggressive and try to preserve the anal sphincter and to avoid permanent ostomies [2]. Nevertheless, the partial or total loss of the rectal reservoir and its replacement with the remaining colon is associated with functional sequelae [4], triggering a negative effect on the pelvic floor region with compromises in evacuation and urinary function. This has been recently investigated and termed as “low anterior resection syndrome” (LARS) [4, 5].

LARS includes a set of symptoms, as well as frequent bowel movements, urgency, fecal incontinence, and disordered evacuation. It was estimated that this syndrome occurs in 70 to 90% of patients [4, 5]. Studies have shown that the main risk factors for developing LARS after surgery are radiotherapy and tumor height, which both have a negative impact on bowel function [6, 7]. LARS can show improvement over the first 2 years, but symptoms persist for longer than 2 years in nearly 60% of patients [8].

There are few studies testing interventions to improve bowel symptoms in patients treated for colorectal cancer, especially those who are submitted specifically to sphincter saving surgeries which are associated with LARS. One retrospective study showed the benefits of biofeedback with balloon rectal training on bowel function, measured with questionnaires and an anorectal manometry [9]. Another recent prospective study included patients who experienced fecal incontinence after sphincter-saving surgery and showed that electrical stimulation and biofeedback improved maximal squeeze pressure and stool frequency and diminished the use of antidiarrheal medications [10].

Two systematic reviews analyzed the effects of pelvic floor rehabilitation on the bowel function of rectal cancer patients after surgery. One of them identified that most studies reported significant improvements in stool frequency, incontinence episodes, severity of fecal incontinence, and patients’ health-related quality of life after pelvic floor training with pelvic floor muscle exercises (PFME) and biofeedback [11]. The other systematic review included studies that used multimodal pelvic floor rehabilitation—pelvic floor muscle training, rectal balloon training, and biofeedback—and showed an improvement regarding continence, stool frequency, and quality of life [12]. However, both systematic reviews concluded that their included studies had some methodological limitations, such as a lack of a non-exposed cohort, a lack of independent blinded assessment, heterogeneous treatment protocols, and a lack of long-term follow-up [11, 12]. So, there is a need for methodologically robust randomized controlled trials to test the effectiveness of pelvic floor rehabilitation with muscle training, balloon rectal biofeedback, and electrostimulation on bowel function and quality of life.

In addition, none of these studies addressed the benefits of delivering pelvic floor interventions prior to, as opposed to following, colorectal cancer treatments. Patients might benefit with one session of pelvic floor pre-rehabilitation including health education and teaching the exercise before surgery. Rehabilitation for cancer patients is a new field and is charged with providing care throughout the course of illness and wellness to maximize potential function and alleviate disability [13]. Recently, rehabilitation services have faced a paradigm shift from a restorative to a more prospective approach that aims at prevention and early intervention to mitigate the impact of disability [13]. Pre-rehabilitation of cancer patients refers to preventive rehabilitation interventions aiming to enhance patient’s functional capacity. It allows patients to better tolerate cancer treatments [14, 15].

### Objectives {7}

Accordingly, the aim of this CAncer of Rectum REhabilitation Trial (CARRET) study is to evaluate the effectiveness of a pre- and post-surgical pelvic floor rehabilitation program on the bowel symptoms, pelvic floor function, and quality of life of rectal cancer patients up to 3 months after their rehabilitation. The secondary objectives are to assess adherence to home-based PFME and attendance to rehabilitation sessions and to perform intra and intergroup comparisons regarding bowel symptoms and anorectal function considering all time points (baseline, before stoma removal, immediately and 3 months and 1 year after pelvic floor rehabilitation).

### Trial design {8}

The trial design is a randomized controlled superiority trial with parallel groups (pelvic floor rehabilitation versus control group), with a blinded evaluator, allocation ratio 1:1.

**Fig. 1:**
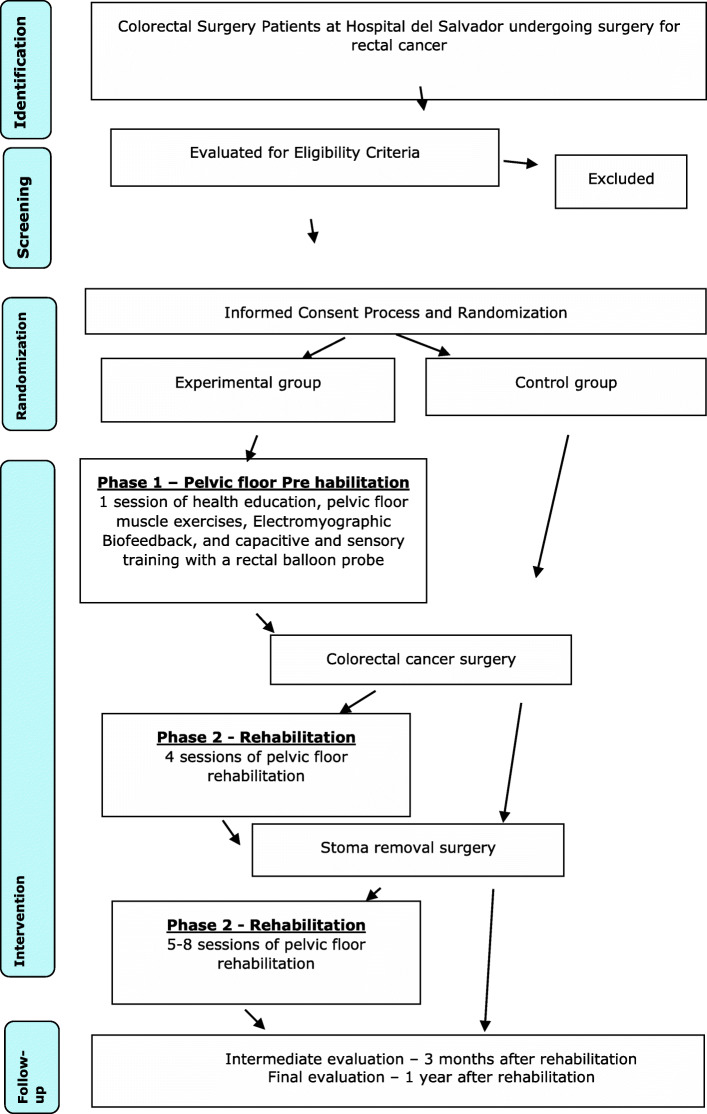
Flow diagram of the study design

Figure 1 summarizes the study design. Patients will be randomized in an experimental group (with pre- and post-surgical pelvic floor rehabilitation + conventional treatment) or control group (only conventional treatment). Both groups will receive 10 sessions of physical exercises before surgery, including aerobic and resistance training [3].

## Methods: participants, interventions, and outcomes

### Study setting {9}

Potential participants will be recruited at Hospital del Salvador, Servicio de Salud Metropolitano Oriente, Santiago de Chile. Evaluations and rehabilitation will be done at the same institution.

### Eligibility criteria {10}

Eligible participants for this study will be adults (aged 18 to 80) with stage I to III rectal cancer who will undergo sphincter preserving surgery at Hospital del Salvador and who have a sufficient understanding of Spanish. They will be recruited at the surgery service for 18 months.

The exclusion criteria will be observed cognitive deficit [mini-mental test score lower than 24] and those patients not meeting criteria for inclusion in the perioperative optimized protocol, such as urgency surgery, neurological diseases like stroke, Parkinson’s or epilepsy, previous gastrectomy, diabetic patient using insulin, renal insufficiency, congestive heart failure, and anesthetic risk ASA 4. Those patients with clinically relevant anastomotic stoma leakage (requiring additional surgical procedures) will be excluded of the study. Neoadjuvant chemoradiotherapy will not be an exclusion criterion.

Interventions will be performed by physical therapists with postgraduation studies on pelvic floor rehabilitation.

### Who will take informed consent? {26a}

The informed consent will be taken from one of the main researchers: CS, LAL, and RS. The process of informed consent will start with a complete explanation of the study, assessments, procedures, risks, and benefits. The patient will have enough time to solve any doubt. Patients will receive a copy of the printed informed consent to carefully read and take it to home for further analysis if they find it necessary.

### Additional consent provisions for collection and use of participant data and biological specimens {26b}

Not applicable.

## Interventions

### Explanation for the choice of comparators {6b}

We decided to compare pre(re)habilitation of the pelvic floor to a control group with standard care without pre(re)habilitation of the pelvic floor because it will give the real benefit of including pelvic floor physiotherapy prior to lower anterior resection for rectal cancer. The study could demonstrate the superiority of starting early pelvic floor rehabilitation.

### Intervention description {11a}

#### Description of the proposed intervention (Fig. 1)

Both groups will receive conventional treatment following institutional perioperative optimized protocol. The pelvic floor intervention for rectal cancer patients will be delivered in two stages: (1) pre-rehabilitation and (2) rehabilitation. Currently, the recommendation is to provide rehabilitation before surgeries in order to prepare patients to improve their functional outcomes. The content of pre-rehabilitation and rehabilitation will include pelvic floor exercises and other techniques aiming at the recovery of pelvic floor maximal function after surgery and the avoidance of low anterior resection syndrome (LARS) symptoms. Pre(re)habilitation will not delay surgical or other treatment times neither interfere in clinical oncological decision-making process.

The pelvic floor intervention, including all stages and techniques, will be provided by a physical therapist with postgraduate studies in pelvic floor treatment and experience in treating rectal cancer patients. This professional will be trained by researchers on the study protocol. The intervention will take place in a private room at Hospital del Salvador.

Stage 1: Pelvic Floor Pre-rehabilitation will be delivered in one session of approximately 40 min with physiotherapist educating on the correct contraction of pelvic floor, teaching pelvic floor muscle exercises, and performing capacitive and sensory training with rectal balloon.

Health education will include the following: the most adequate position for evacuation, self-care strategies such as a high-fiber and low-fat diet that is low in spicy and stimulating food (artificial sweeteners, tea, cola drinks and chocolates), and good bowel habits (the possibility of experiencing an increased urgency to defecate after meal or physical activities).

A booklet was designed for this study with these instructions, and exercises will be provided to the patients as well as an audio that will be sent to their cell phones using the WhatsApp application. The pelvic floor therapist will be given a mobile number with a WhatsApp account so they can send audios and contact patients to reinforce instructions for performing PFME at home. Patients will receive a WhatsApp message once a week to remind them about the exercises. A closer contact with the therapist may increase patients’ self-efficacy and adherence to the treatment [16]. In addition, patients will receive a diary to register the days when they exercise their pelvic floor in order to check adherence to home-based exercises.

Stage 2: Pelvic floor rehabilitation will be delivered in 4 sessions of pelvic floor physiotherapy 3–5 weeks before stoma removal, three times for week. Although anorectal function is not being required for fecal elimination in those with stoma, we will perform PFME and sensory and capacitive training to retrain this function and prevent leakage. In addition, patients will receive 5–8 sessions of pelvic floor physiotherapy starting approximately 2 weeks after stoma removal surgery, over a period of up to 1 month, three times per week, according to patients´ needs. Each session will last approximately 40 min.

Rehabilitation will start around 6–10 weeks after the surgery, depending on physician indication and stoma removal surgery.

The rehabilitation intervention will include pelvic floor exercises, electromyographic biofeedback, and capacitive and sensory training:

Pelvic floor muscle exercises (PFME) will be performed following the protocol of Bø and colleagues [17] who instructed participants to perform a daily total of 24 to 36 slow contractions (high-intensity maximal voluntary contraction with a 6 to 8 s hold). Each slow contraction will be followed by three-to-four fast contractions and then 6 s of rest. In addition, patients will be encouraged to contract the pelvic floor muscles before situations that increase intra-abdominal pressure, known as the “knack” [18]. Patients will be instructed to repeat these exercises at home every day after ostomy removal.

Electromyographic Biofeedback with the equipment Enraf-Nonius Myomed 632X® connected to a large screen for better visualization following a protocol similar to that used by Kuo et al [10] in which neuromuscular stimulation of the pelvic floor muscle was performed 2 to 3 times weekly for a total of 12 treatment sessions. We will use a catheter (Anuform) placed into the anal canal for 10 min during each treatment session. If the patient cannot tolerate the endoanal method, we will use external perineal stimulation. The device delivers a square wave, and ramp up and down time will be set for 2 s with a duration of 8 s at 30 Hz frequency, an on/off time of 1:3, and a pulse duration of 300 ms. Patients will be instructed to actively contract pelvic floor muscles when the electrical stimulus is on.

Capacitive and sensory training with a balloon probe will follow the protocol of Liang et al [9]: a trained therapist will perform repeated inflations and deflations of a balloon in stepwise increments of 5 mL of air or saline solution. The patients will be asked to recognize the volume that induced the urge to defecate and the maximal tolerable volume they were able to hold. The patients will be taught to contract the sphincter in response to the perception of rectal distention. This training will be performed once a week with a duration of approximately 10 min.

Both groups will receive conventional cancer treatment and surgery following perioperative optimized protocols, including 6–10 sessions of multimodal rehabilitation prior to the surgery. It includes physical exercise (aerobic with a stationary bike up to 60–70% of the maximal heart rate reserve calculated with Karnoven et al. (1957) [19] formula and resistance training) and nutritional support. Pre-rehabilitation with physical exercises is recommended by perioperative optimized protocol pathways [3]. A study showed that men with digestive cancers who have a good cardiorespiratory fitness also present a lower risk of mortality [20]. In a large secondary analysis with colorectal cancer patients, authors concluded that multimodal pre-rehabilitation (physical exercise, nutrition, and coping strategies for anxiety) resulted in greater improvement in walking capacity throughout the whole perioperative period when compared to rehabilitation started after surgery [21]. The pre-rehabilitation with physical exercises will be provided by a physical therapist specialized in cancer rehabilitation and trained by researchers on the study protocol. This treatment will be provided at the Service of Physical Medicine and Rehabilitation of Hospital del Salvador.

In case any patient needs adjuvant chemotherapy or radiotherapy, we will wait until the end of these treatments and reassume pelvic floor rehabilitation before ostomy removal. In these cases, patients may take around three additional months to complete treatment.

After completing the follow-up time (3 months) and final evaluations, patients of the control group will be invited to receive 5–10 sessions of pelvic floor rehabilitation.

### Criteria for discontinuing or modifying allocated interventions {11b}

Discontinuation of the intervention will be in the presence of worsening disease condition, upon participant request, or in the presence of any adverse effect.

### Strategies to improve adherence to interventions {11c}

As a strategy to improve adherence, a booklet was designed for this study with instructions, and exercises will be provided to the patients as well as an audio that will be sent to their cell phones using the WhatsApp application. The pelvic floor therapist will be given a mobile number with a WhatsApp account so they can send audios and contact patients to reinforce instructions for performing PFME at home. Patients will receive a WhatsApp message once a week to remind them about the exercises. A closer contact with the therapist may increase patients’ self-efficacy and adherence to the treatment [16]. In addition, patients will receive a diary to register the days when they exercise their pelvic floor in order to check adherence to home-based exercises.

### Relevant concomitant care permitted or prohibited during the trial {11d}

Patients can take institutional routine care, including, if necessary, adjuvant chemoradiotherapy. Only patients of the control group will not be allowed to receive pelvic floor physiotherapy during the study period.

### Provisions for post-trial care {30}

After completing the follow-up time (3 months) and final evaluations, patients of the control group will be invited to receive 5–10 sessions of pelvic floor rehabilitation. No compensation is offered to those who might suffer from harm from trial participation. However, we are negotiating an insurance contract to better protect patients.

### Outcomes {12}

Evaluations will take place at baseline (T1), around 3–5 weeks before stoma removal and before rehabilitation (T2), immediately after rehabilitation (T3), 3 months (T4), and 1 year after rehabilitation (T5).

The primary outcomes are the mean score of bowel symptoms and the mean score of anorectal function. The primary time point will be 3 months after finishing pelvic floor rehabilitation treatment.

#### Main outcome: bowel symptoms

The International Consultation on Incontinence Questionnaire Anal Incontinence Symptoms and Quality of Life Module (ICIQ-B) assess fecal incontinence symptoms (including gas incontinence) and its impact on quality of life. It is separated in three domains with scores from 1 to 21 for bowel patterns, 0–28 for bowel control, and 0–26 for quality of life related to bowel symptoms [22, 23]. It was developed in the UK but still does not have a Spanish version. Its English version has shown to be robust and psychometrically solid, considering analysis of content, construct, criterion validity, internal consistency, and reliability [22, 23]. The questionnaire is in the process of translation to Chilean Spanish and validation by the authors of this study.

In addition, we will use LARS score which is a short questionnaire that has been useful for measuring the impact of intestinal sequelae on the quality of life of patients following sphincter preserving rectal cancer surgery [8]. The LARS score was developed based on a Danish cohort of 961 patients 27 and evaluates incontinence for flatus, incontinence for liquid stools, clustering, and urgency. The score ranges from 0 to 42 and is divided into 0 to 20 (no LARS), 21 to 29 (minor LARS), and 30 to 42 (major LARS). This score has been validated in Spanish [24].

#### Secondary outcomes: anorectal function, quality of life, and pelvic floor muscle function

High-resolution anorectal manometry will be used to assess the maximal resting pressure, maximal squeeze pressure, rectal capacity (maximal tolerable volume), and rectal sensitivity (initial sensation threshold). We will use high-resolution anorectal manometry with a 24-channel water-perfused catheter (Multiplex, Alacer, Biomedica, Sao Paulo, Brazil). This equipment is a low-cost water perfused system which has proven to be adequate for clinical use [25, 26]. According to a previous study, we will define effective improvement as > 15% in the anorectal manometry parameters [9]. This test will be performed by a trained physician.

Quality of life will be evaluated with the validated and widely used instrument of the European Organization for Research and Treatment of Cancer Quality of Life Group (EORTC), the Quality of Life Questionnaire Core-30 (QLQ-C30), and QLQ-CR29 [27–30]. A detailed manual will be used to score each question [31].

Pelvic floor muscle strength will be measured by anal palpation and graded on the Oxford Modified Scale from 0 (no contraction) to 5 (strong). This is the most widely used evaluation on pelvic floor physiotherapy [32].

Adherence to home-based pelvic floor muscle exercises will be measured with an exercise diary. We will record the number of days per week patients performed the exercises following the instructions of the physical therapist. Furthermore, we will record attendance to rehabilitation sessions.

For descriptive purposes, we will use the hospital register of socio-demographic and health-related information, including the following variables: age in years; schooling; self-reported ethnicity; marital status; profession; working status before cancer diagnosis; self-reported comorbidities (diabetes, hypertension, depression, asthma, COPD, smoking, and alcohol consumption); physical activity during leisure time; heart rate, blood pressure; use of anti-motility or anti-diarrhea agents; type of cancer; stage; treatments received (surgery, radiotherapy, chemotherapy, immunotherapy); and body weight, height, and body mass index. SPIRIT guidance: primary, secondary, and other outcomes, including the specific measurement variable (e.g., systolic blood pressure), analysis metric (e.g., change from baseline, final value, time to event), method of aggregation (e.g., median, proportion), and time point for each outcome. Explanation of the clinical relevance of chosen efficacy and harm outcomes is strongly recommended.

### Participant timeline {13}

**Fig. 2:**
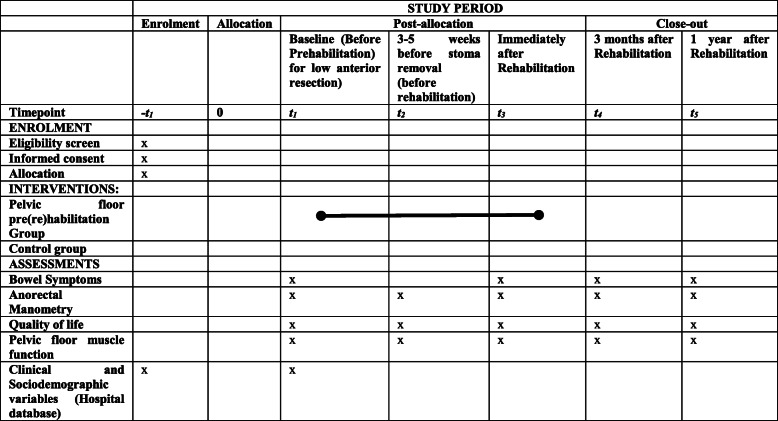
SPIRIT schedule of enrollment, intervention, and assessment

Participant timeline is summarized in SPIRIT (Fig. 2).

### Sample size {14}

The hospital carries out around 50 rectal cancer surgeries per year. The sample size calculation was performed considering the results of a previous pilot study with 10 participants with rectal cancer undergoing pelvic floor rehabilitation [33]. The study used the ICIQ-B and reported results in the domain bowel control after rehabilitation [mean of rehabilitation group = 2.8 (SD = 2.8) and mean of rehabilitation group = 4.9 (SD = 5.7)]. These values were introduced at an online sample size calculator [34] with a power of 80%, alpha 0.05, and an enrollment ratio of 1/1, resulting in an estimated sample size of 56 participants. Then, considering the need of exclusion due to clinically relevant anastomotic leakage, which has been estimated around 10.9% of the cases [35], we will require a total of 62 patients, 31 in each group.

### Recruitment {15}

Recruitment will be performed by the coloproctologist surgeons of the institution who will provide a referral for the study for those patients that comply with eligibility criteria.

## Assignment of interventions: allocation

### Sequence generation {16a}

The allocation of participants to groups will be randomized using computer-randomized allocations prepared by an independent person. Sequence generation will be performed in blocks of 10.

### Concealment mechanism {16b}

Allocation concealment will be guaranteed with sealed opaque envelopes.

### Implementation {16c}

The allocation sequence will be generated by an independent statistician. After screening for eligibility, the participants will be allocated to the next available allocation by the enrolling researcher using sealed and numbered envelopes. The therapist performing the treatments of the intervention group will assign participants to interventions.

## Assignment of interventions: blinding

### Who will be blinded {17a}

The people assessing the outcomes and those analyzing the results/data will be blinded.

### Procedure for unblinding if needed {17b}

Not applicable.

## Data collection and management

### Plans for assessment and collection of outcomes {18a}

One of the researchers, a physical therapist specialized in cancer rehabilitation, will be responsible for contacting patients to assess outcomes at baseline and at follow-up visits. All included questionnaires are validated. Anorectal manometry will be performed by another of the researchers, a physician well-trained in the technique.

### Plans to promote participant retention and complete follow-up {18b}

Patients will receive a WhatsApp message once a week to remind them about the exercises. In case they do not use this app, the therapist will call them once a month. A closer contact with the therapist may increase patients’ self-efficacy and adherence to the treatment [16].

### Data management {19}

Data will be tabulated in an Excel sheet anonymously using codification by a research assistant. Control and experimental group will be coded anonymously with numbers (1, 2) aiming to blind the person who will perform statistical analysis. Data will be stored in the secured network of Universidad Bernardo O´Higgins with access to the researchers and, for security reasons, in an external hard drive which will be used to back up regularly the database. The data monitoring team will be the researcher that assesses outcomes and another responsible for data analysis.

### Confidentiality {27}

Personal information will be maintained only by the therapist in a special folder stored at the institution where data will be collected.

### Plans for collection, laboratory evaluation, and storage of biological specimens for genetic or molecular analysis in this trial/future use {33}

Not applicable because there are no biological specimens in this study.

## Statistical methods

### Statistical methods for primary and secondary outcomes {20a}

Data will be maintained in printed questionnaires which will be registered on an Excel dataset. A monitor will check the completeness of data and rigorously control patient’s follow-up. Data will be analyzed with descriptive (mean, standard deviation, median, interquartile range, and frequencies) and inferential statistics at the program SPSS®. A normality test will be performed using Kolmogorov-Smirnov criteria. For variables which follow normality criteria, we will use an independent T test to compare groups. In the case of a non-normal distribution, we will use the Mann-Whitney U test. To compare variables between pre- and post-intervention, we will use a paired T test or a Wilcoxon test. A significance threshold of *p* < 0.05 will be adopted for all tests.

### Interim analyses {21b}

Not applicable.

### Methods for additional analyses (e.g., subgroup analyses) {20b}

Subgroup analysis will be performed for gender groups.

### Methods in analysis to handle protocol non-adherence and any statistical methods to handle missing data {20c}

In cases of lost to follow-up rates lower than 20%, we will perform an intention-to-treat analysis, using single imputation procedures with the mean of the group to replace missing values.

### Plans to give access to the full protocol, participant level-data, and statistical code {31c}

In case of audits of health authorities and funder or other researchers needing to access data for scientific purposes, we will give access to the full protocol, participant level-data, and statistical code.

## Oversight and monitoring

### Composition of the coordinating center and trial steering committee {5d}

Not applicable.

### Composition of the data monitoring committee, its role and reporting structure {21a}

This clinical trial will not include a data monitoring external committee because it includes therapies widely used by pelvic physical therapists as well as assessments commonly applied among rectal cancer patients. Nonetheless, we will establish an internal committee for monitoring preliminary data regarding safety. This committee will include the researchers that assess the main outcomes and the main researcher. They will perform constant preliminary data analysis looking for safety and effectiveness. In case of any reported adverse event, this committee will ask for analysis and recommendation of an external surgeon with expertise in treating rectal cancer. Data monitoring group is independent from the funder and sponsor.

### Adverse event reporting and harms {22}

Possible adverse events might include excessive bleeding or pain. The first will be evaluated with a question asking for the frequency of anal bleeding during the last month (never, once a week or less, twice or three times a week, once a day, many times on a day, continually). In addition, the amount of bleeding will be quantified with the cup measure (less than half a cup, half a cup, more than half a cup, a cup, and so on). They will be assessed immediately after pelvic floor rehabilitation and immediately after pelvic floor pre-rehabilitation. Excessive pain will be determined as a pain equal to or higher than 7 on a visual analog scale from 0 (no pain) to 10 (the worst pain).

### Frequency and plans for auditing trial conduct {23}

Auditing will be conducted only in case the funder or Ethical Committee requires.

### Plans for communicating important protocol amendments to relevant parties (e.g., trial participants, ethical committees) {25}

In case of the need of protocol modifications (e.g., changes to eligibility criteria, outcomes, analyses), we will communicate it to relevant parties (which are the Ethical Committee and trial registry).

### Dissemination plans {31a}

The results of this study will be published at scientific journals and in scientific international/national activities.

## Discussion

This paper presents a protocol for a prospective randomized controlled trial to study the effectiveness of pelvic floor pre(re)habilitation both prior to and after a low anterior resection for rectal cancer, versus no rehabilitation at all, on bowel symptoms, pelvic floor function, and quality of life. There is another similar study protocol published, but as it is a feasibility study, their primary outcome measure is the proportion of eligible patients approached who consented to and attended the educational session. The secondary outcomes included patient compliance, the acceptability of the intervention (assessed using qualitative interviews) and pelvic floor tone, patient bowel function, and quality of life [36]. Besides that, two systematic reviews showed promises of the benefits of PFME on bowel function after rectal cancer. However, the methodological limitations of the included studies justify the need of randomized controlled trials testing the effectiveness of pelvic floor rehabilitation to prevent LARS [11, 12].

This study is innovative because it will include prehabilitation, i.e., a session of pelvic floor physiotherapy prior to the surgery. A possible limitation of this study will be related to oncological results of the included patients, for example if the patient requires adjuvant chemotherapy and have other complications, will delay rehabilitation, which might affect the results. Furthermore, the long follow-up period added to by the social unrest in Chile that started in 2019 and COVID-19 outbreak in early 2020 might increase attrition rates or delay the study results.

The proposed research received ethics approval from Servicio de Salud Metropolitano Oriente [Orient Metropolitan Health Service], Santiago de Chile (approval date: October 08, 2019). Published results are expected in 2022.

## Trial status

Trial recruitment is not started yet due to delays caused by coronavirus pandemics. This is the first protocol version registered on 21 January 2020. We plan to start recruitment by 15 January 2021 and to complete recruitment by 30 December 2022.

## Data Availability

The copy of the booklet for patients will be available upon request to the corresponding author. Data will be available for researchers who provide a methodologically sound proposal for meta-analysis beginning 3 months following the main results’ publication. Data can be obtained by contacting the main researcher. Data will be available by contacting by email the main researcher using details on ANZCTR or trial published articles. Email for requests: cinara.sacomori@ubo.cl or csacomori@yahoo.com.br.

## Abbreviations

CARRET: Cancer of Rectum Rehabilitation Trial
LARS: Low anterior resection syndrome
PFME: Pelvic floor muscle exercises

## References

[1] Bray F, Ferlay J, Soerjomataram I, Siegel RL, Torre LA, Jemal A (2018). Global cancer statistics 2018: GLOBOCAN estimates of incidence and mortality worldwide for 36 cancers in 185 countries. CA Andom J Clin.

[2] Trenti L, Galvez A, Biondo S, Solis A, Vallribera-Valls F, Espin-Basany E, Garcia-Granero A, Kreisler E (2018). Quality of life and anterior resection syndrome after surgery for mid to low rectal cancer: a cross-sectional study. Eur J Surg Oncol..

[3] Gustafsson UO, Scott MJ, Hubner M, Nygren J, Demartines N, Francis N, Rockall TA, Young-Fadok TM, Hill AG, Soop M, de Boer HD, Urman RD, Chang GJ, Fichera A, Kessler H, Grass F, Whang EE, Fawcett WJ, Carli F, Lobo DN, Rollins KE, Balfour A, Baldini G, Riedel B, Ljungqvist O (2019). Guidelines for perioperative care in elective colorectal surgery: Enhanced Recovery After Surgery (ERAS) Society Recommendations: 2018. World J Surg.

[4] Hain E, Manceau G, Maggiori L, Mongin C, Prost à la Denise J, Panis Y (2017). Bowel dysfunction after anastomotic leakage in laparoscopic sphincter-saving operative intervention for rectal cancer: a case-matched study in 46 patients using the Low Anterior Resection Score. Surg..

[5] Van der Heijden JAG, Thomas G, Caers F, van Dijk WA, Slooter GD, Maaskant-Braat AJG (2018). What you should know about the low anterior resection syndrome – clinical recommendations from a patient perspective. Eur J Surg Oncol..

[6] Croese AD, Whiting S, Vangaveti VN, Ho YH (2018). Using sacral nerve modulation to improve continence and quality of life in patients suffering from low anterior resection syndrome. ANZ J Surg..

[7] Sun W, Dou R, Chen J, Lai S, Zhang C, Ruan L, Kang L, Deng Y, Lan P, Wang L, Wang J (2018). Impact of long-course neoadjuvant radiation on postoperative low anterior resection syndrome and quality of life in rectal cancer: post hoc analysis of a randomized controlled trial. Ann Surg Oncol..

[8] Sarcher T, Dupont B, Alves A, Menahem B (2018). Anterior resection syndrome: what should we tell practitioners and patients in 2018?. Journal of Visceral Surgery.

[9] Liang Z, Ding W, Chen W, Wang Z, Du P, Cui L (2016). Therapeutic evaluation of biofeedback therapy in the treatment of anterior resection syndrome after sphincter-saving surgery for rectal cancer. Clin Colorectal Cancer..

[10] Kuo LJ, Lin YC, Lai CH, Lin YK, Huang YS, Hu CC, Chen SC (2015). Improvement of fecal incontinence and quality of life by electrical stimulation and biofeedback for patients with low rectal cancer after intersphincteric resection. Arch Phys Med Rehabil..

[11] Lin K-Y, CL G, Denehy L, Frawley H. (2015). Pelvic floor muscle training for bowel dysfunction following colorectal cancer surgery: a systematic review. Neurol Urodynamics..

[12] Visser WS, Riele WW, Boerma D, Van Ramshorst B, Van Westreenen HL (2014). Pelvic floor rehabilitation to improve functional outcome after a low anterior resection: a systematic review. Ann Coloproctol.

[13] Gerber LH, Hodsdon B, Comis LE, Chan L, Gallin J, McGarvey CL (2017). Contemporary issues in cancer rehabilitation a brief historical perspective of cancer rehabilitation and contributions from the National Institutes of Health. PM&R..

[14] Gillis C, Li C, Lee L, Awasthi R, Augustin B, Gamsa A, Liberman AS, Stein B, Charlebois P, Feldman LS, Carli F (2014). Prehabilitation versus rehabilitation. Anesthesiol..

[15] Treanor C, Kyaw T, Donnelly M (2018). An international review and meta-analysis of prehabilitation compared to usual care for cancer patients. J Cancer Surviv..

[16] Sacomori C, Berghmans B, Mesters I, de Bie R, Cardoso FL (2015). Strategies to enhance self-efficacy and adherence to home-based pelvic floor muscle exercises did not improve adherence in women with urinary incontinence: a andomized trial. J Physiother..

[17] Bø K, Talseth T, Holme I (1999). Single blind, randomized controlled trial of pelvic floor exercises, electrical stimulation, vaginal cones, and no treatment in management of genuine stress incontinence in women. BMJ..

[18] Miller JM, Ashton-Miller JA, Delancey JO (1998). A pelvic muscle pre-contraction can reduce cough-related urine loss in selected women with mild SUI. J Am Geriatr Soc..

[19] Karvonen MJ, Kentala E, Mustala O (1957). The effects of training on heart rate; a longitudinal study. Ann Med Exp Biol Fenn.

[20] Peel JB, Sui X, Matthews CE, Adams SA, Hébert JR, Hardin JW, Church TS, Blair SN (2009). Cardiorespiratory fitness and digestive cancer mortality: findings from the aerobics center longitudinal study. Cancer Epidemiol Biomarkers Prev.

[21] Minnella EM, Bousquet-Dion G, Awasthi R, Scheede-Bergdahl C, Carli F (2017). Multimodal prehabilitation improves functional capacity before and after colorectal surgery for cancer: a five-year research experience. Acta Oncol (Madr)..

[22] Cotterill N, Norton C, Avery KNL, Abrams P, Donovan JL (2008). A patient-centered approach to developing a comprehensive symptom and quality of life assessment of anal incontinence. Dis Colon Rectum..

[23] Donovan J, Bosch JLHR, Gotoh M, Abrams P, Cardozo L, Khoury S, Wein A (2005). Symptom and quality of life assessment. Incontinence.

[24] Juul T, Ahlberg M, Biondo S, Emmertsen KJ, Espin E, Jimenez LM, Matzel KE, Palmer G, Sauermann A, Trenti L, Zhang W, Laurberg S, Christensen P (2014). International validation of the low anterior resection syndrome score. Ann Surg.

[25] Silva RMB, Herbella FAM, Gualberto D (2018). Normative values for a new ando-perfused high resolution manometry system. Arq Gastroenterol.

[26] Viebig RG, Franco JTY, Araujo SV, Gualberto D (2018). Water-perfused high-resolution anorectal manometry (hram-wp): the first andomize study. Arq Gastroenterol..

[27] Aaronson NK, Ahmedzai S, Bergman B, Bullinger M, Cull A, Duez NJ, Filiberti A, FlechtnerH FSB, deHaes JC (1993). The European Organization for Research and Treatment of Cancer QLQC30: a quality of-life instrument for use in international clinical trials in oncology. J Natl Cancer Inst.

[28] Irarrázaval ME, Rodríguez PF, Fasce G, Silva F, Waintrub H, Torres C (2013). Calidad de vida en cáncer de mama: Validación del cuestionario QLQ-BR23 en Chile. Rev Med Chile.

[29] Arraras JI, Suárez J, de la Vega FA, Vera R, Asín G, Arrazubi V, Rico M, Teijeira L, Azparren J (2011). The EORTC quality of Life questionnaire for patients with colorectal cancer: EORTC QLQ-CR29 validation study for Spanish patients. Clin Translational Oncol..

[30] Whistance RN, Conroy T, Chie W, Costantini A, Sezer O, Koller M, Johnson CD, Pilkington SA, Arraras J, Ben-Josef E, Pullyblank AM, Fayers P, Blazeby JM, European Organisation for the Research and Treatment of Cancer Quality of Life Group (2009). Clinical and psychometric validation of the EORTC QLQ-CR29 questionnaire module to assess health-related quality of life in patients with colorectal cancer. Eur J Cancer.

[31] Fayers P, Aaronson N, Bjordal K, Groenvold M, Curran D, Bottomley A (2001). EORTC QLQ-C30 Scoring Manual.

[32] Laycock J, Jerwood D (2001). Pelvic floor muscle assessment: the PERFECT scheme. Physiother..

[33] Lin KY, Denehy L, Frawley HC, Wilson L, Granger CL (2018). Pelvic floor symptoms, physical, and psychological outcomes of patients following surgery for colorectal cancer. Physiother Theory Pract..

[34] Clincalc. Sample size calculator. Retrieved from https://clincalc.com/Stats/SampleSize.aspx on 10th February 2019.

[35] Rodríguez-Ramírez SE, Uribe A, Ruiz-García EB, Labastida S, Luna-Pérez P (2006). Risk factors for anastomotic leakage after preoperative chemoradiation therapy and low anterior resection with total mesorectal excision for locally advanced rectal cancer. Rev Invest Clín.

[36] Powell-Chandler A, Rees B, Broad C, Torkington J, O'Neill C, Cornish JA, PARiS (Physiotherapy and Anterior Resection Syndrome) Trial Management Group (2018). Physiotherapy and Anterior Resection Syndrome (PARiS) trial: feasibility study protocol. BMJ Open.

